# Biocompatibility and osseointegration properties of a novel high strength and low modulus β- Ti10Mo6Zr4Sn3Nb alloy

**DOI:** 10.3389/fbioe.2023.1127929

**Published:** 2023-02-10

**Authors:** Jiantao Liu, Kao Wang, Xingyuan Li, Xiwei Zhang, Xi Gong, Yihan Zhu, Zhiwei Ren, Bin Zhang, Jun Cheng

**Affiliations:** ^1^ Department of Orthopedics, The First Affiliated Hospital of Xi’an Jiaotong University, Xi’an, Shaanxi, China; ^2^ Xi’an Jiaotong University, Xi’an, Shaanxi, China; ^3^ Medical School of Yan’an University, Yan’an, Shaanxi, China; ^4^ Institute of Translational Medicine, Shenzhen Second People’s Hospital, Shenzhen, China; ^5^ Northwest Institute for Nonferrous Metal Research, Shaanxi Key Laboratory of Biomedical Metal Materials, Xi’an, China

**Keywords:** titanium alloys, biocompatibilities, osseointegrations, osteoblasts, biomaterials

## Abstract

**Introduction:** Ti6Al4V titanium alloy is widely used in producing orthopedic and maxillofacial implants, but drawbacks include high elastic modulus, poor osseointegration performance, and toxic elements. A new medical titanium alloy material with better comprehensive performance is urgently needed in the clinic.

**Methods:** Ti10Mo6Zr4Sn3Nb titanium alloy (referred to as Ti-B12) is a unique medical *ß* titanium alloy material developed by us. The mechanical properties of Ti-B12 depict that it has advantages, such as high strength, low elastic modulus, and fatigue resistance. In our study, the biocompatibility and osseointegration properties of Ti-B12 titanium alloy are further studied to provide theoretical guidance for its clinical transformation.

**Results and Discussion:** The titanium alloy Ti-B12 displays no significant effect on MC3T3-E1 cell morphology, proliferation, or apoptosis *in vitro*. Neither Ti-B12 titanium alloy nor Ti6Al4V titanium alloy depicts a significant difference (*p* > 0.05); Ti-B12 material extract injected into the abdominal cavity of mice does not cause acute systemic toxicity. The skin irritation test and intradermal irritation test reveal that Ti-B12 does not cause skin allergic reactions in rabbits. Compared to Ti6Al4V, Ti-B12 titanium alloy material has more advantages in promoting osteoblast adhesion and ALP secretion (*p* < 0.05). Although there is no significant difference in OCN and Runx2 gene expression between the three groups on the 7th and 14th days of differentiation induction (*p* > 0.05), the expression of Ti-B12 group is higher than that of Ti6Al4V group and blank control group. Furthermore, the rabbit *in vivo* test present that 3 months after the material is implanted in the lateral epicondyle of the rabbit femur, the Ti-B12 material fuses with the surrounding bone without connective tissue wrapping. This study confirms that the new β-titanium alloy Ti-B12 not only has low toxicity and does not cause rejection reaction but also has better osseointegration performance than the traditional titanium alloy Ti6Al4V. Therefore, Ti-B12 material is expected to be further promoted in clinical practice.

## 1 Introduction

For decades, Ti6Al4V has been one of the most widely used materials for orthopedics and oral implants due to its lightweight, corrosion resistance, fatigue resistance, and non-toxic and non-magnetic properties (Trevisan et al., 2018; Xi and Wong, 2021). However, clinical studies have found complications, such as loosening and displacement of the Ti6Al4V implants (Liu et al., 2019; Gkiatas et al., 2021). The reason is closely related to the defect of Ti6Al4V (Jing et al., 2020; Zhan et al., 2020; Zheng et al., 2020). Firstly, as compared to cortical bone (17–20 GPa) and cancellous bone (about 4 GPa), Ti6Al4V has an elastic modulus of about 110 GPa (Murr, 2017). A higher elastic modulus will cause a “stress shielding” effect (Pehlivan et al., 2020), causing excessive stress on the local cortical bone and low stress on the underlying bone, leading to osteolysis. Second, Ti6Al4V is a bioinert material without biological activity and osteoinducibility, and it is challenging to osteointegrate with surrounding bone tissues after implantation in the human body (Backes et al., 2021; Su et al., 2021). Additionally, these alloys use two toxic elements, V and Al. Studies have confirmed that V even shows higher toxicity than Cr and Ni and will accumulate in various organs after long-term implantation, which can induce cancer (Mello et al., 2019). However, the element Al enters the human body in the form of Al salt, which causes organ damage and side effects such as bone softening, nervous system disorder, and anemia (Willis et al., 2021). Al element may be related to Alzheimer’s disease (Vojdani, 2021).

Because of the defects of Ti6Al4V materials, domestic and foreign researchers have been committed to researching and developing new medical titanium alloys with better comprehensive properties (Sarraf et al., 2022; Szczesny et al., 2022). In the mid-1990s, Switzerland and Germany successively developed the second generation of medical titanium alloys Ti6Al7Nb and Ti5Al2.5Fe, which replaced V with Nb and Fe. However, these two alloys still contain an Al element that has adverse reactions to the human body, and the elastic modulus is still as high as 4∼10 times that of bone tissue. The mismatch between the mechanical properties of implant and bone tissue was unsolved. Recently, researchers have begun to focus on new *ß* titanium alloys (Nadammal et al., 2022; Sing, 2022; Vonavkova et al., 2022). This material can retain the unstable phase, such as the sub-stable *ß* phase or martensite structure, at room temperature, giving the material good processing plasticity and toughness. Adjusting the microstructure of material through post-processing, heat treatment, strength, toughness, elastic modulus, corrosion resistance, wear resistance, and fatigue properties can be significantly enhanced (Sidhu et al., 2021).

Additionally, Zr, Mo, Sn, Ti, Ta, Nb, Pd, Hf, and other non-toxic elements to human tissue are used in the new β-type titanium alloy to improve biocompatibility (Sidhu et al., 2021). The United States, Japan, Russia, and other countries have made some achievements in the research and development of biomedical β-type titanium alloys, such as Ti12Mo6Zr2Fe, TiNbTaZr, and Ti51Zr18Nb. These β-type titanium alloys have been successively applied in the medical field.

Using non-toxic elements, we developed a novel *ß* type of biomedical titanium alloy -- Ti10Mo6Zr4Sn3Nb, referred to as Ti-B12, and obtained the national invention patent (ZL.201110184053.X) (Cheng et al., 2020). Mechanical tests demonstrate that when the elastic modulus of Ti-B12 titanium alloy is as low as 50 GPa, the tensile strength is about 970 MPa, and the elongation can reach more than 40%, which has obvious advantages over pure Ti. The elastic modulus is about 60.7 GPa when the tensile strength reaches 1,010 MPa, while the elongation of material can still reach 15%, which is much less than Ti6Al4V. Additionally, Ti-B12 titanium alloy can obtain high strength, low modulus, and excellent comprehensive mechanical properties that match degree through the ‘martensite transition’ and the intermediate transition phase "ω phase” in the process of processing and heat treatment. Hence, it shows good biomechanical compatibility under static conditions. The Ti-B12 titanium alloy still has a high fatigue strength limit (σN ≥500 MPa, the number of cycles is 10^7^) and recoverable strain (εmax-R≤2.25%) after cyclic loading and fatigue test, which is conducive to the implant material maintaining its original excellent biomechanical compatibility under long-term dynamic loading conditions. The results of this study illustrate that the biocompatibility of Ti-B12 titanium alloy is superior to that of Ti6Al4V material. However, it has better bone integration properties and is an excellent medical titanium alloy material, which is expected to achieve clinical transformation in the future.

## 2 Methods

### 2.1 Statement of animal rights

Procedures for the care and use of animals were approved by the Ethics Committee of the First Hospital of Xi’an Jiaotong University (2022-196). All applicable institutional and governmental regulations concerning the ethical use of animals were followed.

### 2.2 Alloy design and composition

**FIGURE 1:**
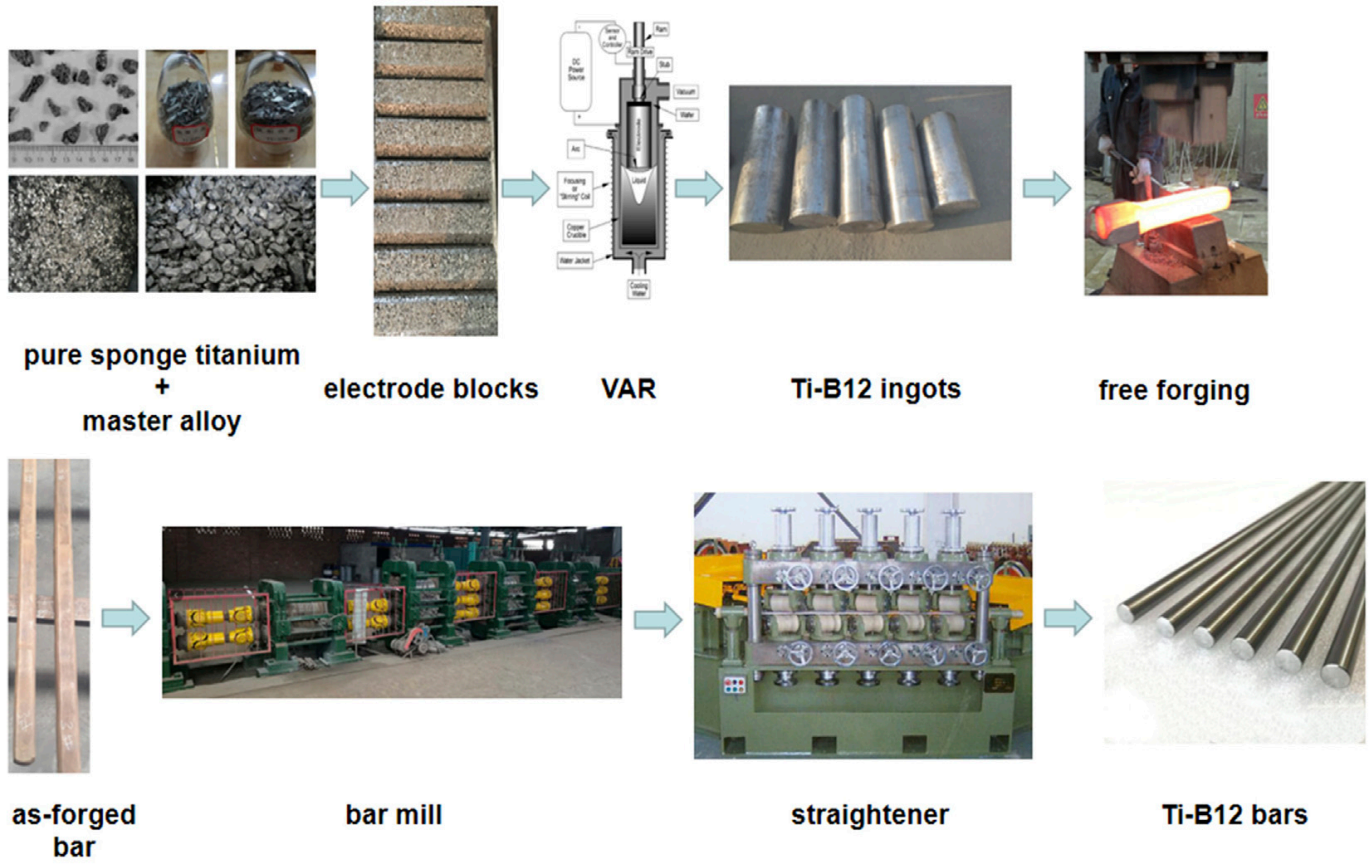
Synthesis process of Ti-B12 titanium alloy.

The biomedical metastable β-type titanium alloy with independent intellectual property rights of the Northwest Institute of Non-ferrous Metals was selected as the experimental material. Zero-grade small-particle sponge titanium, industrial grade sponge zirconium (Zr-1), Ti-32Mo, Ti-60Sn, and Nb-47Ti intermediate alloys were selected for three times melting by consumable vacuum arc melting furnace to ensure the uniform chemical composition of alloy ingot and avoid the occurrence of composition segregation and high and low-density inclusion. The measured results of chemical composition of Ti-B12 alloy ingot are as follows: Mo: 10.1%, Zr: 6.05%, Sn: 4.11%, Nb: 2.98%, O: 0.19%. The transus temperature is about 765 °C by conventional quenching metallography. Under the condition of 1,000 °C, using a 1-ton air-free forging hammer, the alloy ingot was upset and pulled many times to break the coarse cast structure, and finally, the square bar of 50 × 50×L mm was obtained. Under 850 °C, the transverse pass mill was used to roll the bar. After peeling and straightening, the alloy bar with a diameter of 8 mm was finally obtained. The synthesis process is shown in Figure 1.

### 2.3 Materials preparation

Using a numerical control lathe and Edm wire cutting equipment, a disc with a diameter of 8 mm and thickness of 1 mm and a cylinder with a diameter of 5 mm and height of 10 mm were manufactured, which were used for the comprehensive evaluation experiment of biocompatibility and bone integration performance. Ti6Al4V was used as the control material. All samples were cleaned by ultrasonic wave with distilled water, acetone, 95% ethanol, and distilled water in turn, each step for 30 min. The above operation was repeated three times. The cleaned samples were autoclaved at 121 °C for 40 min, dried in a vacuum, and put into sterile tubes for subsequent cell and animal experiments.

### 2.4 Biocompatibility of Ti-B12

#### 2.4.1 Cell morphology observation and proliferation experiment

The sterilized Ti-B12 and Ti6Al4V materials were placed in sterile Petri dishes, and the growth medium containing α-MEM medium (Cellmax), 10% FBS (MRC), and 1% penicpstreptomycin mixture (Solarbio) was added and placed in the cell incubator for 72 h to prepare the extract. MC3T3-E1 cells in the logarithmic growth phase were collected and seeded in 96-well cell culture plates at 1×10^5^ mL^-1^ (100 μL per well). They were divided into three groups: A, B, and C, with five wells set at each time point in each group. The culture medium was changed after 24 h in the cell incubator. Groups A and B were added with 200 μL of Ti-B12 and Ti6Al4V material extract, respectively. Group C was added with an ordinary growth medium, which was changed every 3 days. At 1, 3, and 7 days, cell growth status was observed by microscope and photographed, and then cell proliferation rate was detected by the Cell Counting Kit 8 (CCK8) kit (Biyuntian, Shanghai, China).

#### 2.4.2 Cell apoptosis assay

MC3T3-E1 cells in good growth state were inoculated into sterile 6-well plates containing TI-B12 and Ti6Al4V, respectively, at the concentration of 1 × 10^5^ mL^-1^, and each well was inoculated with 3 mL. In the blank control group, only osteoblasts were inoculated with three multiple wells in each group, and cells were cultured in the cell incubator for 24 h. Annexin V-PE/7-AAD Apoptosis Detection Kit (Yeasen) was used to detect cell apoptosis by flow cytometry.

#### 2.4.3 Acute systemic toxicity test

A total of twelve healthy adult male mice were randomly assigned to three groups, Ti-B12 group, Ti6Al4V group, and control group, each consisting of four mice. The ethics committee of Xi, a Jiaotong University, approved the experiment. According to the systemic toxicity detection method of national standard GBT 16886.11-1997, mice in Ti-B12 group and Ti6Al4V group were intraperitoneally injected with metal material normal saline extract (50 mL kg^-1^), while mice in the control group were intraperitoneally injected with normal saline (50 mL kg^-1^). Status, body weight, toxic reactions, and the number of dead mice were observed daily for 5 days after injection. According to its manifestations, acute systemic toxicity can be classified into non-toxic, mildly toxic, moderately toxic, severely toxic, and death. Table 1 illustrates the specific evaluation indicators.

**TABLE 1:** Evaluation criteria for systemic toxicity.

Toxicity level	Performance of toxicity
Non-toxic	No symptoms of toxicity were observed
Mild poisoning	The symptoms were mild, without reduced movement or dyspnea
Moderate poisoning	Symptoms of abdominal irritation were obvious, with less exercise, dyspnea, drooping eyelids, diarrhea, and significant weight loss to 15–17 g or 20% ± 3% of body weight loss
Severe poisoning	Respiratory failure, cyanosis, tremors, severe abdominal irritation. Eyelids droop, and weight drops to less than 15 g
Death	Death after injection

#### 2.4.4 Skin irritation test

Six adult male New Zealand white rabbits, weighing 2.5–3 kg, were provided and raised by the Laboratory Animal Center of Xi’an Jiaotong University. Before the experiment, hair on both sides of the rabbit spine was removed (about 10 × 15 cm), and the two materials were placed in the hair removal area on both sides of the spine, respectively. The material surface was covered with 2.5 × 2.5 cm gauze, soaked with 0.9% normal saline, and fixed with bandages for 4 h. After removing the materials, the skin conditions at the contact sites on both sides of the skin were continuously observed for 1, 48, and 72 h, and the type of skin irritation response was evaluated according to the scoring system (Tables 2, 3).

**TABLE 2:** Skin and intradermal stimulation response scoring system.

Type	0	1	2	3	4
Erythema and eschar	No erythema	Very subtle erythema	Clear erythema	Moderate erythema	Severe erythema (purple-red) to eschar formation
edema	No edema	Very subtle edema	Clear edema (swelling, not beyond the edge of area)	Moderate edema (swelling about 1 mm)	Severe edema (swelling of more than 1 mm, beyond the range of application)

**TABLE 3:** Type of stimulus-response.

Reaction type	Very mild	Mild	Moderate	Severe
The average score	0–0.4	0.5–1.9	2.0–4.9	5.0–8.0

#### 2.4.5 Intradermal stimulation test

After sterilization, Ti-B12 and Ti6Al4V materials were placed into sterile six-well plates, 0.9% normal saline (polar extract) and olive oil (non-polar extract) were added, and placed in an incubator for 72 h to obtain the material extract. Six adult male New Zealand white rabbits, weighing 2.5–3 kg, were provided by the Laboratory Animal Center of Xi’an Jiaotong University. Before the experiment, the hair on both sides of the rabbit spine (about 10 × 15 cm) was removed, and six injection points were set on both sides. The test material saline extract and normal saline were injected at six intradermal injection points before and after one side of each rabbit spine. Olive oil extract of the same material and olive oil was injected at six intradermal injection points before and after the other side. Skin conditions at each injection site were recorded at 1, 48, and 72 h after injection and the types of intradermal stimulation responses were evaluated according to the scoring system (Tables 2, 3).

### 2.5 Osteointegrative properties of Ti-B12

#### 2.5.1 Osteoblast adhesion ability

Cell adhesion was detected by acridine orange staining and cell counting. MC3T3-E1 cells at a concentration of 1×10^5^ mL^-1^ were seeded in sterile 24-well plates containing Ti-B12 and Ti6Al4V, respectively, with 1 mL per well. Acridine orange staining was performed at 1, 3, and 5 h of co-culture, respectively. Three re-wells were set for each material and each time point.

#### 2.5.2 Osteoblast adhesion status

MC3T3-E1 cells in the logarithmic growth phase were seeded in sterile 12-well plates with Ti-B12 and Ti6Al4V materials at a concentration of 1×10^5^ mL^-1^ and incubated at 37 °C, 95% relative humidity, and 5% CO_2_ for 72 h. Before scanning electron microscopy, the culture was terminated for fixation, dislocation, critical point drying, and gold spraying.

#### 2.5.3 Detection of IL-6 secretion

MC3T3-E1 cells were seeded at a concentration of 1×10^5^ mL^-1^ were seeded in 24-well plates containing Ti-B12, the Ti6Al4V group, and the control group. Each time point of each group was set with three re-holes. Cells were cultured in the cell incubator for 2, 4, and 6 days, respectively. At the end of culture, 10 μL of cell culture medium was taken to detect the concentration of IL-6 according to the interleukin 6 (IL-6) ELISA kit (Biyuntian, Shanghai, China).

#### 2.5.4 Alkaline phosphatase activity

MC3T3-E1 cells in the logarithmic growth phase were digested in a cell suspension and seeded in a 24-well plate containing two types of materials at a concentration of 1×10^5^ mL^-1^. The blank control group contained no materials. Three re-wells were set for each time point in each group. The cells were incubated at 37 °C and 5% CO_2_ for 1, 3, and 5 days, respectively. At the time of termination of culture, 10 μL of cell culture medium sample was taken to detect ALP concentration according to the instruction of ALP ELISA kit (Biyuntian, Shanghai, China).

#### 2.5.5 Expression of osteogenic related genes

The growth medium was replaced with an osteogenic differentiation medium after 24 h in the Ti-B12, Ti6Al4V, and blank control groups, and the growth medium was replaced every 3 days. After 7 and 14 days of induction, the culture medium was carefully sucked and discarded, the branch samples were washed twice with PBS, RNA was extracted, and osteogenic-related genes (ALP, OCN, and Runx2) were detected by RT-PCR. Table 4 illustrates the primer sequences.

**TABLE 4:** The upper and lower primer sequences.

Gene	The upper primer sequences	The lower primer sequences
ALP	5′-gag​gtc​aca​tcc​atc​ctg​cgc​tgg-3′	5′-gag​tac​cag​tcc​cga​tcg​gcc​gag-3′
OCN	5′-ctg​ctc​act​ctg​ctg​acc​ctg​gct-3′	5′-gct​ttg​tca​gac​tca​ggg​ccg​ctg-3′
Runx2	5′-gca​aca​aga​ccc​tgc​ccg​tgg-3′	5′-gaa​act​ctt​gcc​tcg​tcc​gct​ccg-3′
GAPDH	5′-atc​act​gcc​acc​cag​aag​ac-3′	5′-gtg​agt​ttc​ccg​ttc​agc​tc-3′

#### 2.5.6 Osteoblast mineralization

MC3T3-E1 cells were seeded in 12-well plates containing Ti-B12, Ti6Al4V, or not at a cell concentration of 1×10^5^ mL^-1^. After 24 h, the medium was changed to osteoblast mineralization medium (α-mem medium containing 10% FBS, 10 mmol/L β-sodium glycerophosphate, 10 nmol/L dexamethasone, and 50 mg/L vitamin C). The mineralized medium was changed every 3 days. After 30 days of culture, the medium was discarded, and the bottom of well and the material were washed three times with PBS, fixed with 4% paraformaldehyde for 30 min, and stained with 40 mmol.L^-1^ alizarin red for 20 min. Calcified nodules after staining were observed under an inverted microscope. Finally, 10% cetylpyridine chloride was added to dissolve mineralized nodules, and absorbance was detected at a wavelength of 590 nm.

#### 2.5.7 *In vivo* experiments on animals

**FIGURE 2:**
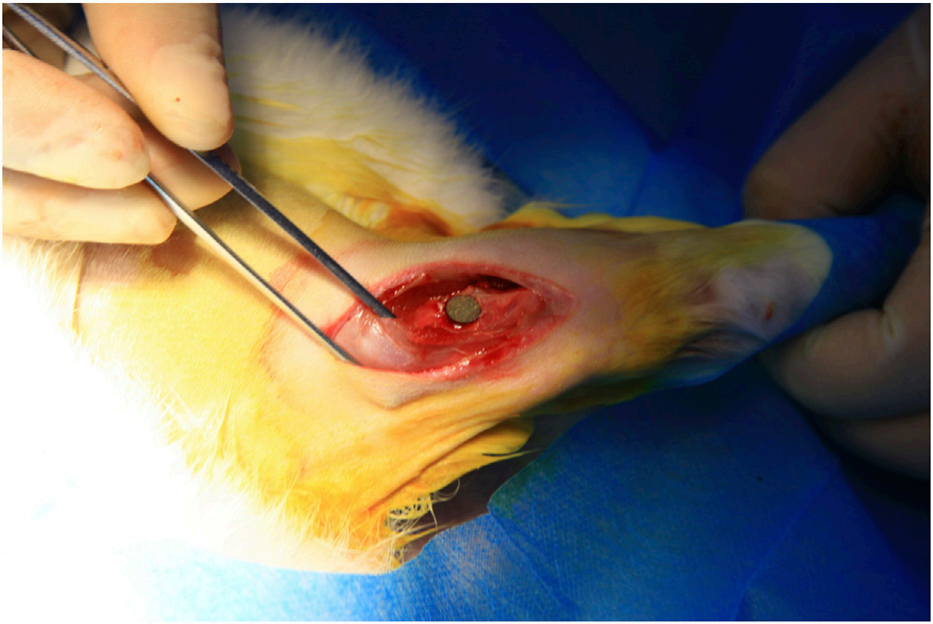
The prostheses were implanted in the lateral epicondyle of the rabbit femur.

Ten adult male New Zealand white rabbits weighing 2.5–3 kg were divided into two groups, group A was implanted with Ti-B12 material, and group B was implanted with Ti6Al4V material. After administration of pentobarbital through the ear vein, the epicondyle of both rabbit femurs of rabbits was exposed, and a hole was made perpendicular to the femur with a 2.0 g Schner wire. The sterilized material was implanted and sutured layer by layer (Figure 2). Penicillin (10,000 U.kg^-1^) was used to prevent infection 3 days after the operation, and an X-ray examination was performed 3 months after the procedure. Three months after the operation, the animals were sacrificed by overdose of anesthesia to obtain specimens for hard tissue sections to observe bone growth around the material. The production process of hard tissue section is rough as follows: the femur specimen containing titanium alloy is made into a resin block after formalin fixation, gradient alcohol dehydration, and resin immersion. A diamond band saw was used to cut the resin blocks into sheets with a thickness of 200–300 μm, and the sheets were cleaned and adhered to the resin slides. The sample was ground to a thickness of approximately 50 µm using an EXAKT tissue mill (E400CS), and the sample surface was polished and stained with toluidine blue. The prepared sections were observed under a microscope.

### 2.6 Statistical analysis

Data were expressed as mean ± standard deviation (SD) and analyzed statistically using one-way analysis of variance (ANOVA). Differences between the two groups and each time point were analyzed by independent sample *t*-test. The significance level of data is set at *p* < 0.05.

## 3 Results

### 3.1 Biocompatibility of Ti-B12

#### 3.1.1 Cell morphology observation and proliferation experiment

**FIGURE 3:**
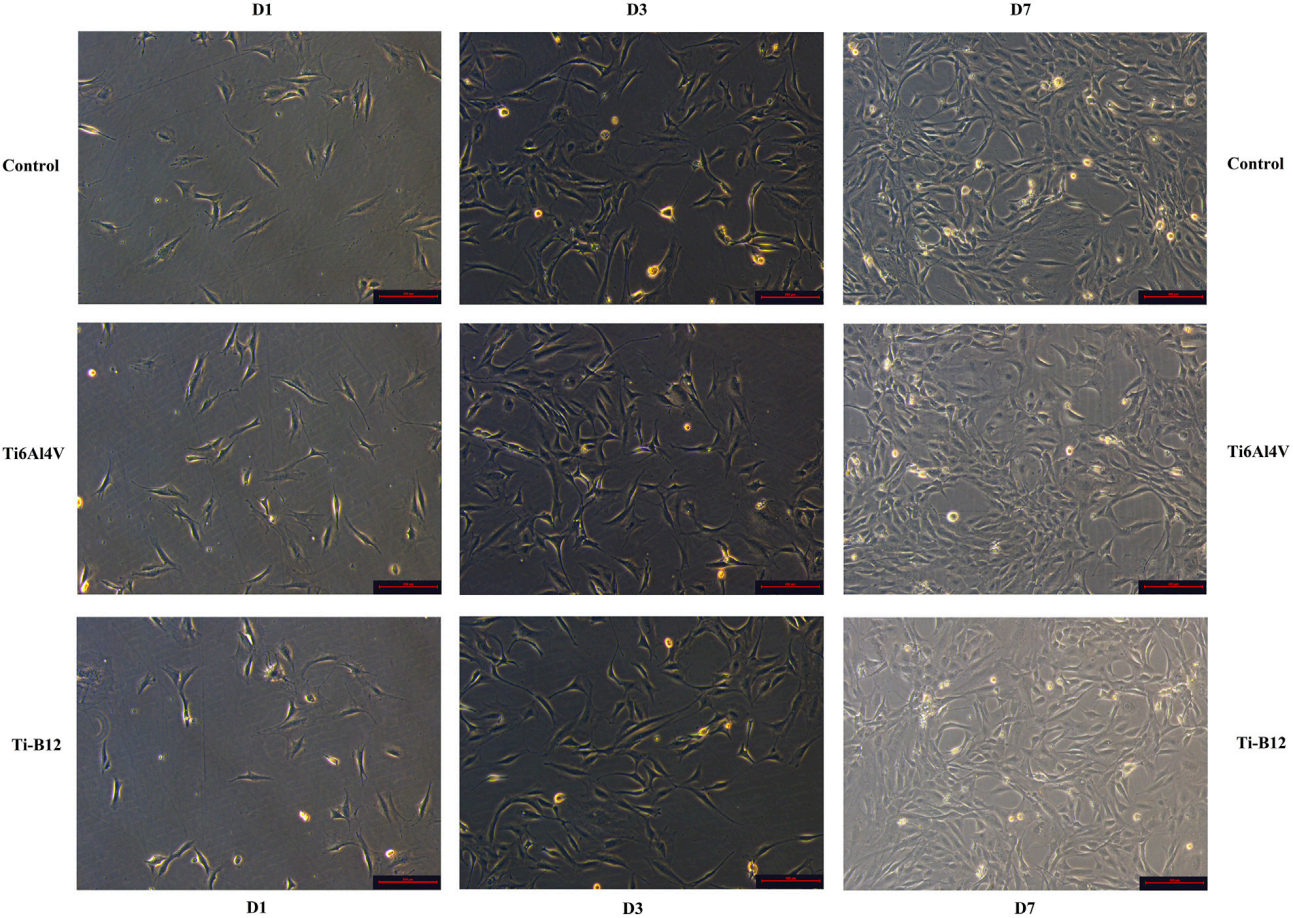
MC3T3-E1 Cell morphology observation of three groups on the 1^st^, 3^rd^, and 7^th^ days.

MC3T3-E1 cells grew well in Ti-B12 material extract, Ti6Al4V extract, and blank control group, but no significant differences in cell morphology were observed at each time point. On the first day, the cells adhered to the wall and grew in spindle shape or dendritic shape. On the third day, the cells increased significantly, grew rapidly, and the refractive index was strong. On the seventh day, the cells were in good condition, with entire cytoplasm and an increased number, almost covering the bottom of pore (Figure 3).

**FIGURE 4:**
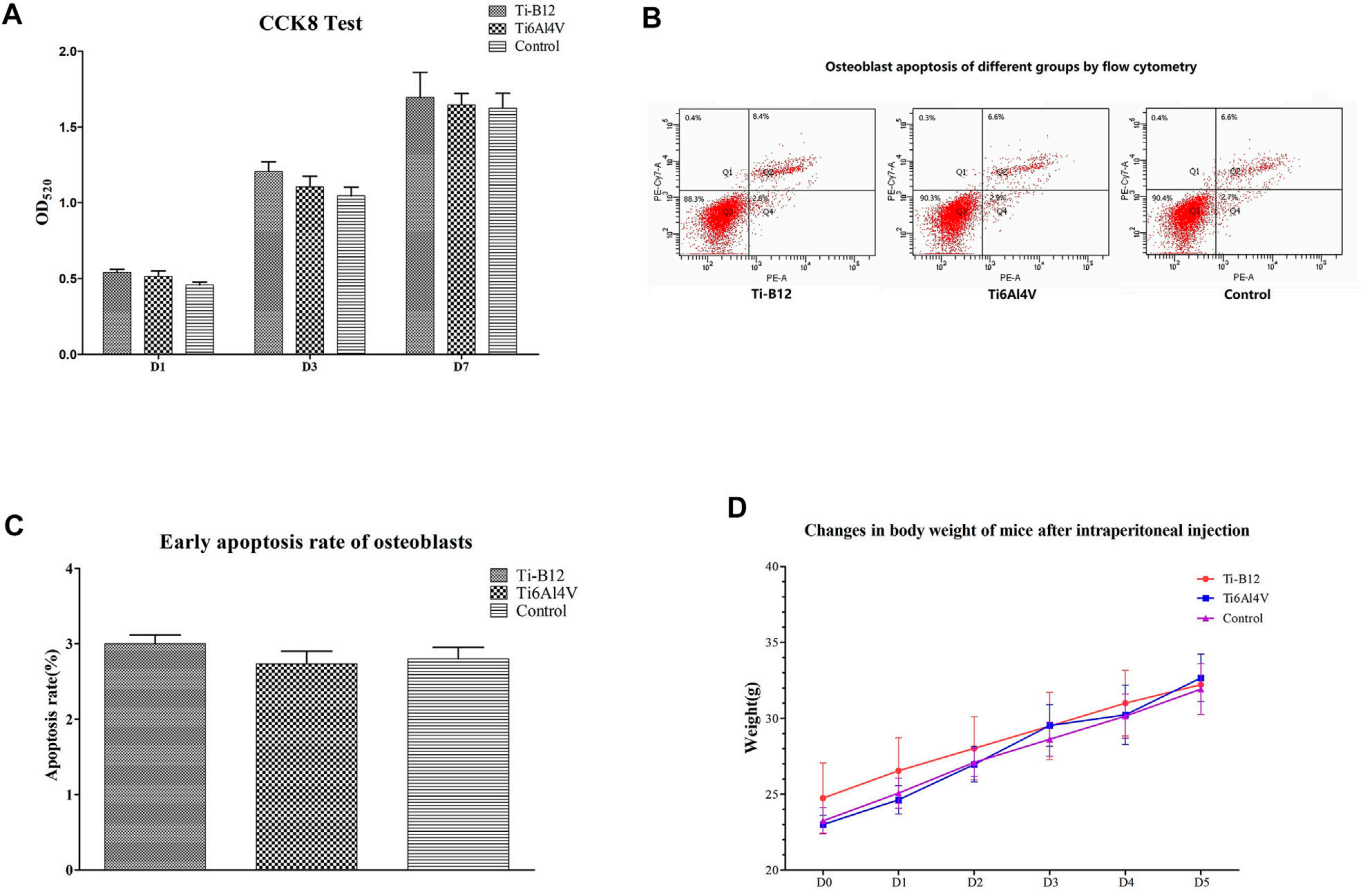
Ti-B12 biocompatibility test *in vitro*. **(A)** MC3T3-E1 cell proliferation assay by CCK8. **(B)** cell apoptosis assay by flow cytometry. **(C)** comparison of early apoptosis rate among three groups. **(D)** weight changes in three groups of mice.

Figure 4A presents that cells in the three groups proliferated with the increase in culture time. Although the Ti-B12 group demonstrated a higher proliferation rate than the Ti6Al4V and blank control groups, there was no significant difference (*p* > 0.05). Therefore, MC3T3-E1 cells can proliferate normally in Ti-B12 material extract, which meets the cytotoxicity requirements of biomedical materials and products.

#### 3.1.2 Cell apoptosis assay

Figure 4B displays the flow cytometry detection results, where quadrants B1, B2, B3, and B4 depict the proportion of necrotic cells, middle and late apoptotic cells, viable cells, and early apoptotic cells in all cells, respectively. Statistical analysis of the early apoptosis rate of three groups found that the early apoptosis rate of the Ti-B12 group, the Ti6Al4V group, and the blank control group was similar (Figure 4C), and there was no statistical difference (*p* > 0.05). The results revealed that Ti-B12 alloy did not induce early apoptosis of osteoblasts and had good biological activity.

#### 3.1.3 Acute systemic toxicity test

All mice were observed after injection. Within 5 days, the weight of mice in the three groups increased continuously (Figure 4D), and no symptoms of peritoneal irritation, respiratory depression, decreased movement, cyanosis, diarrhea, tremor, or death occurred. It can be concluded that Ti-B12 meets the requirements of biological materials and products.

#### 3.1.4 Skin irritation test

**FIGURE 5:**
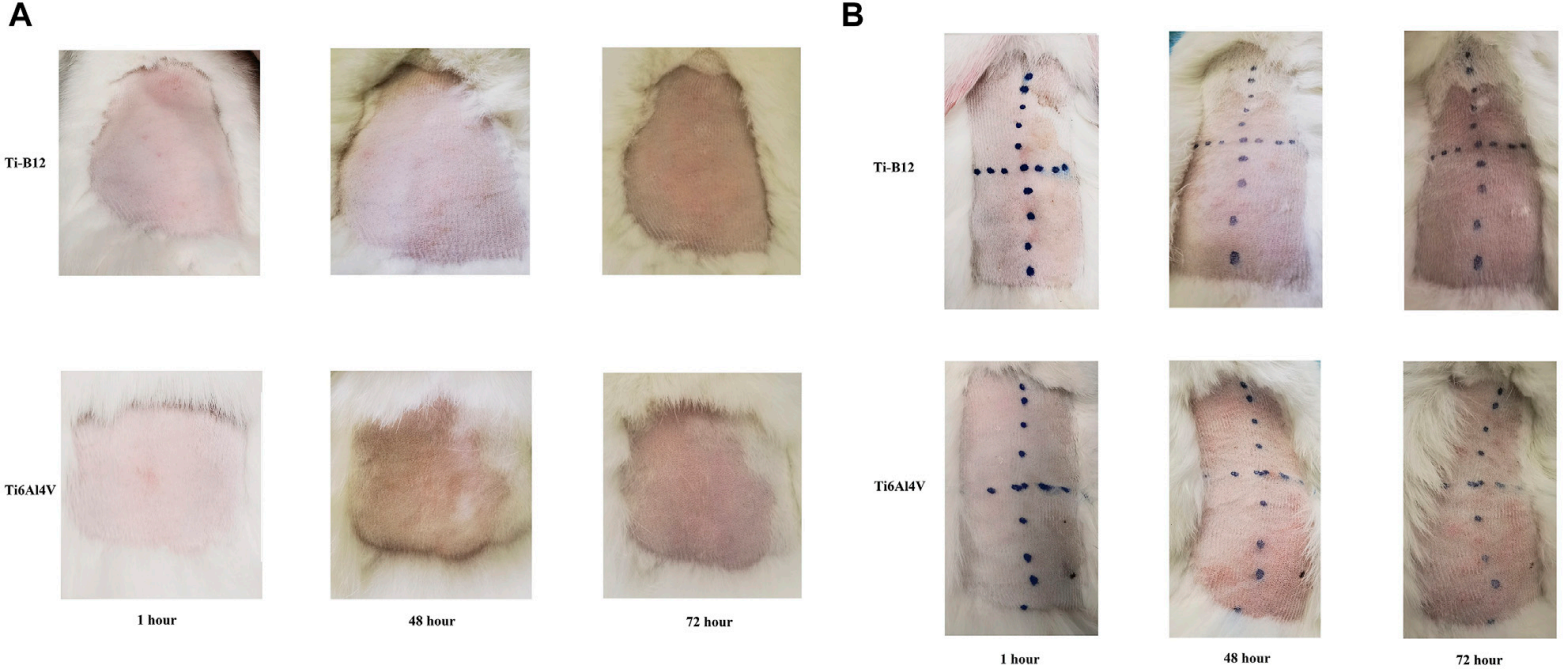
**(A)** skin irritation test. **(B)** intradermal stimulation test.

Figure 5A presents that erythema, eschar, and edema did not appear in the application site of Ti-B12 and Ti6Al4V materials in 1, 48, and 72 h, and their scores were all 0. The type of stimulation reaction was very mild, which met the requirements of the skin irritation experiment with biological materials and products.

#### 3.1.5 Intradermal stimulation test

Figure 5B presents that the skin at the injection site demonstrated no erythema, eschar and edema at 1, 48, and 72 h after intradermal injection of polar and non-polar extracts of Ti-B12 and Ti6Al4V materials; this was the same as the performance after the intradermal injection of polar and non-polar control solvents, with an average score of 0; this indicates that Ti-B12 meets the requirements of subcutaneous stimulation of biological materials and products.

### 3.2 Osteointegrative properties of Ti-B12

#### 3.2.1 Osteoblast adhesion ability

**FIGURE 6:**
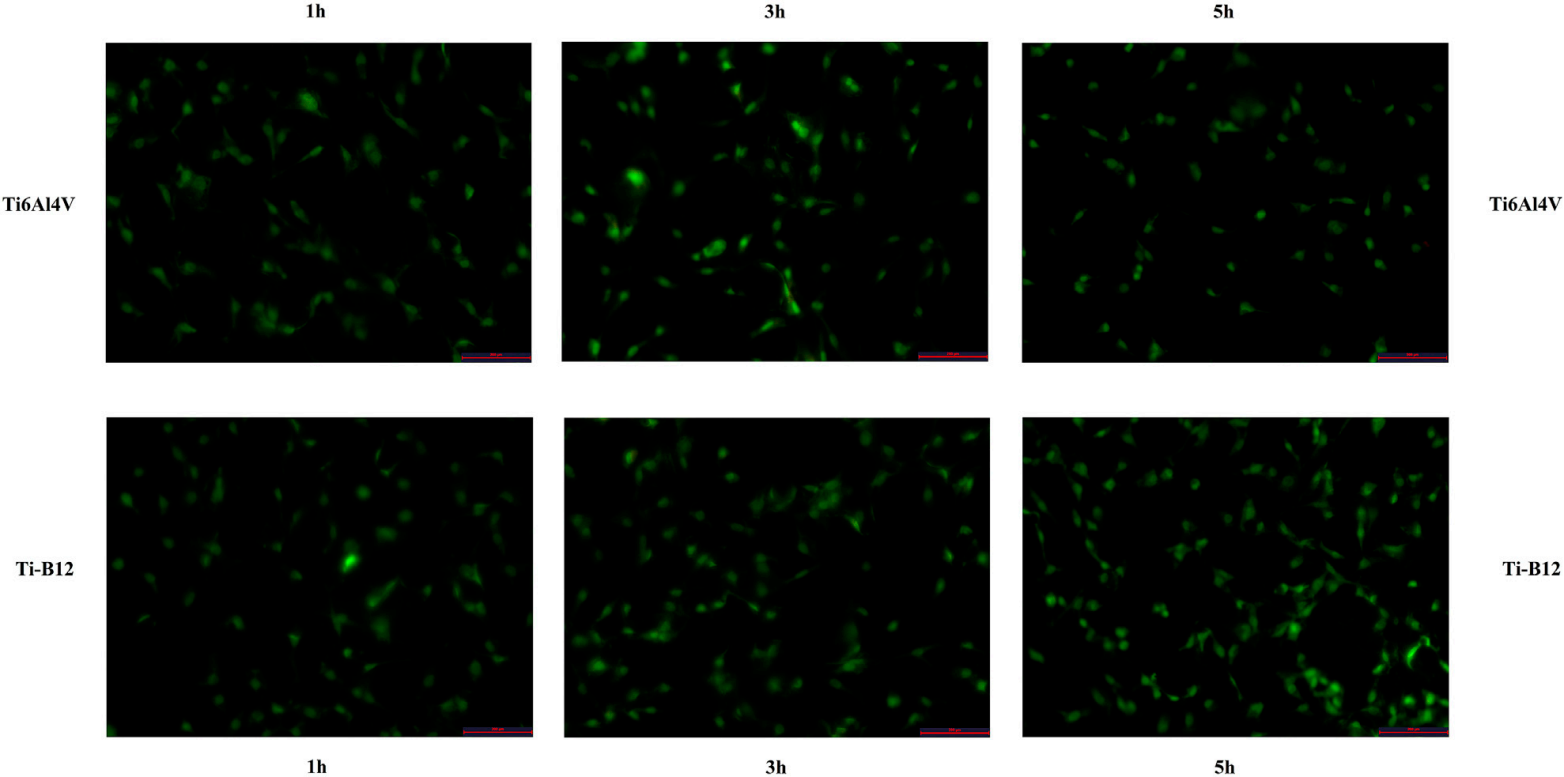
The adhesion ability of MC3T3-E1 on the surface of osteoblasts of two materials was observed under a fluorescence microscope.

The number of adherent cells on the surface of two materials was counted under a fluorescence microscope (Figure 6), and the number of adherent cells of Ti-B12 and Ti6Al4V increased with the extension of culture time. At different times, the number of adherent cells on the surface of Ti-B12 material was higher than that of Ti6Al4V material. It can be seen that Ti-B12 has a better adhesion capacity for osteoblasts.

#### 3.2.2 Osteoblast adhesion status

**FIGURE 7:**
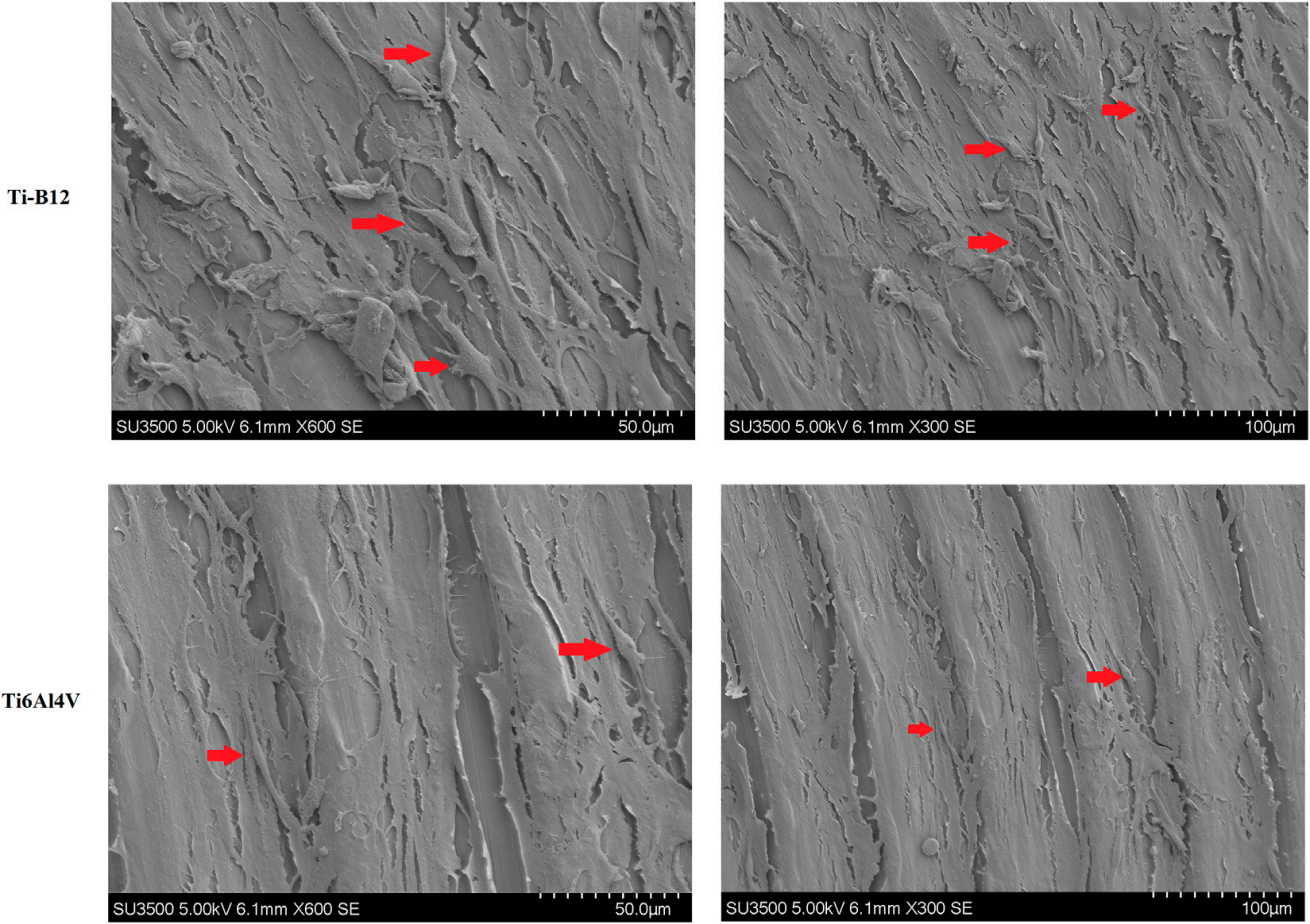
Observation by scanning electron microscope of MC3T3-E1 cells on the surface of Ti-B12 and Ti6Al4V.

Scanning electron microscope observation of cells on the surface of Ti-B12 and Ti6Al4V demonstrated that a large number of MC3T3-E1 cells were attached to the surface of the material, which was elongated and spindle-shaped, with many pseudopodia, and the growth status of the cells was good (Figure 7). The number and morphology of Ti-B12 adhesion cells were significantly better than that of Ti6Al4V, indicating that Ti-B12 material was more conducive to osteoblast adhesion.

#### 3.2.3 Detection of IL-6 secretion

**FIGURE 8:**
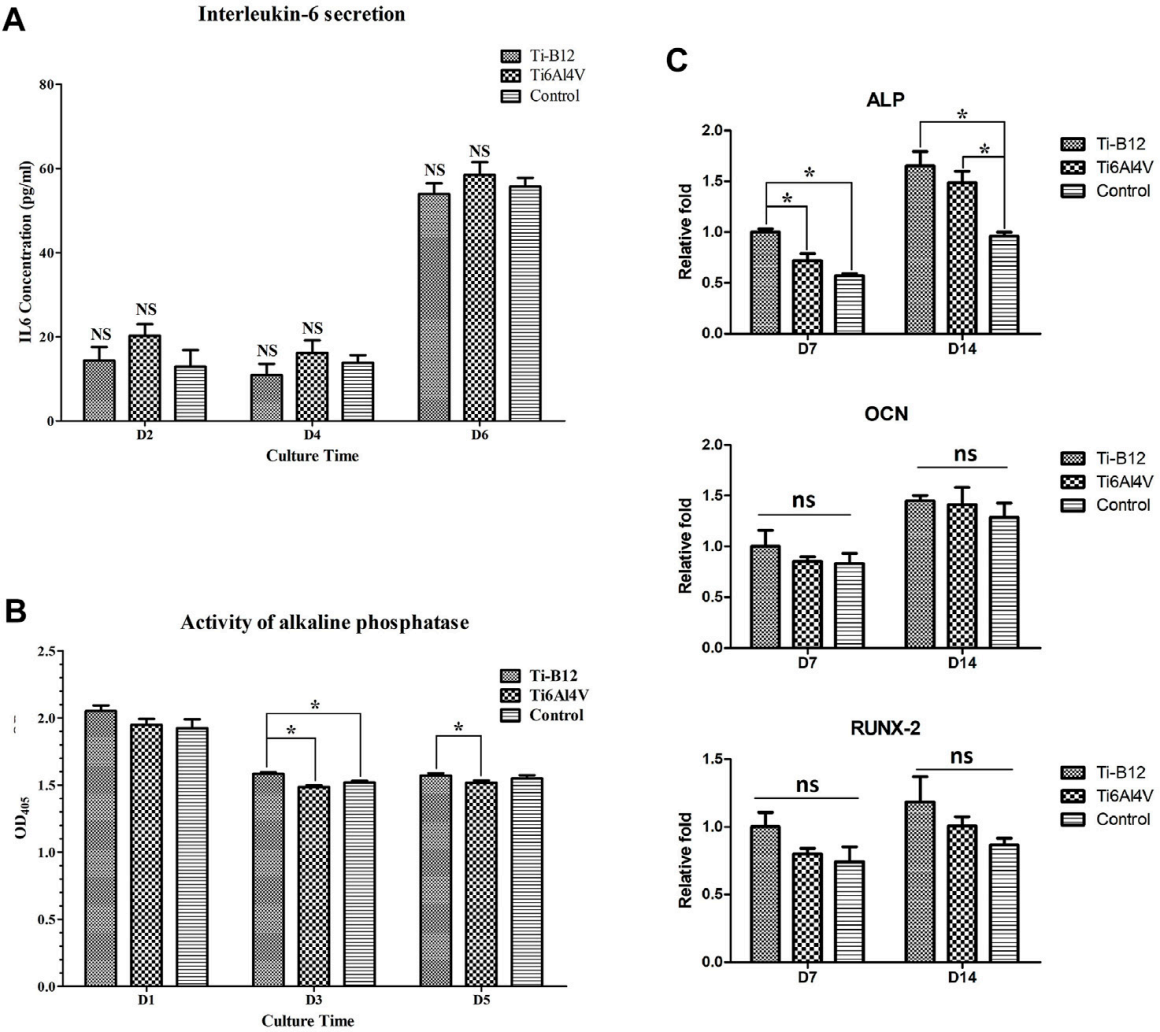
Detection of osteogenic properties *in vitro*. **(A)** detection of IL-6 secretion. **(B)** comparison of alkaline phosphatase activity. **(C)** expression of osteogenic-related genes.

The IL-6 content in the supernatant of the three groups was compared and analyzed at each time point. As we can see from the results (Figure 8A), there was no significant difference in IL-6 secretion between the two alloys and the blank control group at 2, 4, and 6 days of culture (*p* > 0.05); this indicates that the Ti-B12 titanium alloy does not stimulate osteoblast secretion of IL-6 and does not increase the immunogenic response.

#### 3.2.4 Alkaline phosphatase activity

Compared to the Ti6Al4V group (Figure 8B), the expression of osteoblast alkaline phosphatase in the Ti-B12 group was significantly increased on the third and fifth day of culture (*p* < 0.05) but also significantly higher than the blank control group on the third day (*p* < 0.05), indicating that the Ti-B12 alloy had a better promotion effect on early differentiation of osteoblasts.

#### 3.2.5 Expression of osteogenic related genes

Figure 8C presents that on the seventh day of differentiation induction, ALP gene expression in Ti-B12 group was highest in the three groups (*p* < 0.05). ALP gene expression in Ti-B12 group was higher than that in the blank control group on day 14 of induced differentiation (*p* < 0.05). Although there were no significant differences in the expression of the OCN and Runx2 gene between the three groups at 7 and 14 days after differentiation induction (*p* > 0.05), the expression of the OCN and Runx2 gene in the Ti-B12 group was higher than that in the Ti6Al4V group and the blank control group.

#### 3.2.6 Osteoblast mineralization

**FIGURE 9:**
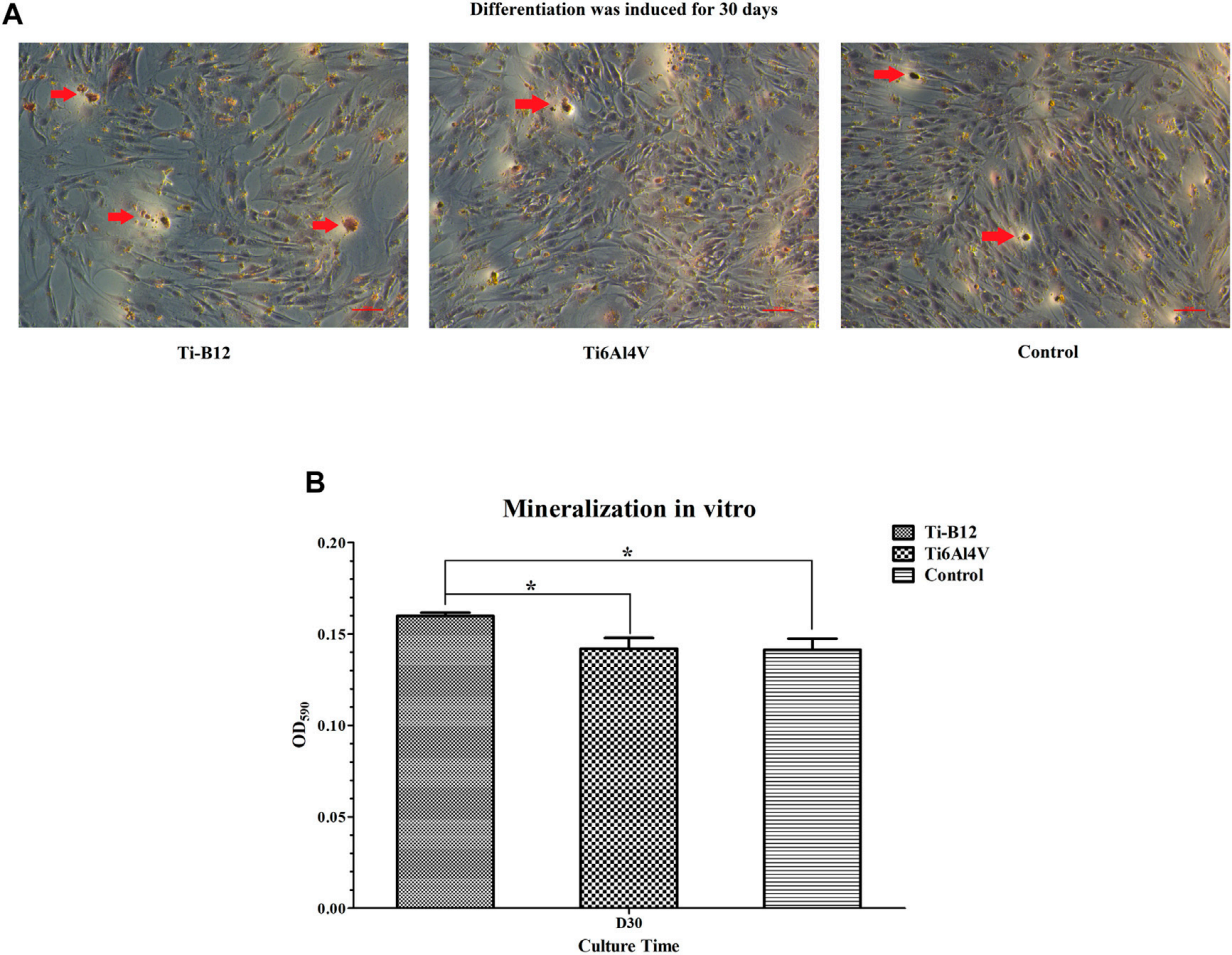
Osteoblast mineralization. **(A)** calcium salts in mineralized nodules were stained dark red by alizarin red staining, shown by the red arrow. **(B)** quantitative determination of calcium salt deposition.

The results revealed that the deposition of calcium salt was significantly more in the Ti-B12 group than in the Ti6Al4V group and the blank control group (*p* < 0.05), and there was no significant difference between the latter two groups (*p* > 0.05), indicating that Ti-B12 material has a better promotion effect on osteoblast mineralization *in vitro* (Figures 9A, B).

#### 3.2.7 *In vivo* experiments on animals

**FIGURE 10:**
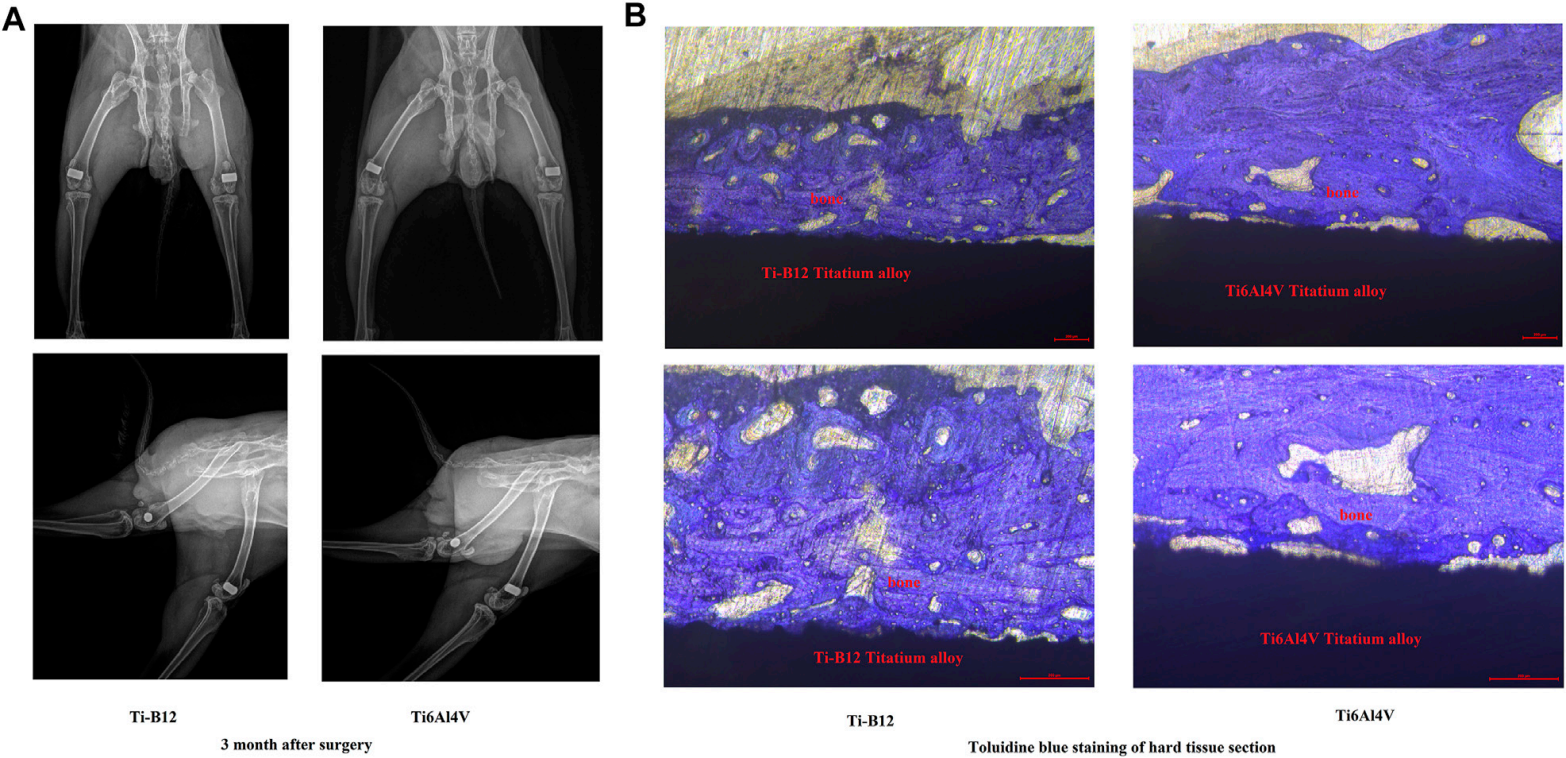
*In vivo* experiments on animals. **(A)** results of the X-ray examination 3 months after the operation; **(B)** results of the toluidine blue staining of hard tissue sections.

Figure 10A displays the X-ray examination results 3 months after the operation, showing that both alloys were perfectly embedded in the lateral epicondyle of femur, and no peeling or displacement was found. The samples were well integrated with the surrounding bone, and no osteoporosis or inflammation was observed. Figure 10B presents the toluidine blue staining results of hard tissue sections of experimental rabbits 3 months after implantation of different alloys into the lateral epicondyle of femur. It was observed that the two alloys were closely bound to bone and that no connective tissue was found at the junction between the material and bone. However, the contact rate of bone tissue around Ti-B12 titanium alloy is better than that of Ti6Al4V.

## 4 Discussion

Titanium alloys are widely used in orthopedic implants due to their good biocompatibility and mechanical properties (Kaur and Singh, 2019). However, some studies illustrated that Ti6Al4V (α+β titanium alloy), commonly used in clinical practice, has the drawbacks of excessive elastic modulus and toxic elements (Ottria et al., 2018). Therefore, many scholars have researched to exploit titanium-based materials with non-toxic and mechanical properties matching natural bone (Bedouin et al., 2019; Shi et al., 2021). To achieve this goal, the effects of different β-stabilizers such as Ta, Zr, Mo, and Nb on the properties of tailored titanium alloys have attracted the great attention of researchers (Mendes et al., 2016; Zhao et al., 2018; Zhang et al., 2021). These β-stabilizers have good shape memory effects, superelasticity, and a low Young modulus in titanium alloys. It can be seen from the recently published literature that the development of β-titanium alloy materials without Al and V elements has been a research hot spot (Kaur and Singh, 2019). Our group developed a new β-titanium alloy Ti-B12 with high strength and low elastic modulus using Mo, Zr, Sn, and Nb β stabilizers. Preliminary mechanical tests have also confirmed that the new material has excellent mechanical properties. However, the ultimate goal of customized β-Ti alloys is to obtain alloy properties similar to the physiological properties of tissues, which requires the new materials to have excellent mechanical properties, as well as excellent biocompatibility and osseointegration properties. Therefore, this study aims to identify the biological properties of Ti-B12 titanium alloy and provide theoretical guidance for its application in orthopedic implants.

Using biological materials replaces or repairs living tissues to enhance organ and tissue functions and treat diseases. The premise of its function is implantation in the human body. Therefore, in addition to providing specific functions, good biocompatibility is the basic condition that biomaterials must meet (Rahmati and Mozafari, 2019; Shahi et al., 2019). Biocompatibility refers to the reaction of medical materials and human tissues when interacting (Apostu et al., 2018). After the biological material is implanted into the human body, the implant is subjected to the fatigue of body fluids and various external forces. The material components will enter the human body due to corrosion or wear. Biocompatibility in cells means that the material has little or no cytotoxicity and does not cause cell necrosis or apoptosis. In the body, the local rejection of the contact site is small and does not cause local congestion, edema, erythema, and other inflammatory and allergic reactions. The overall response of tested animals is good, and no systemic symptoms such as agitation, anorexia, or even death occur. *In vivo* and *in vitro* experiments are essential components of the biocompatibility evaluation of medical materials. Using *in vivo* experiments, the biocompatibility of material is evaluated by inflammation, allergy, and other reactions of body tissue. The advantage of *in vivo* experiments is that the whole organism is used as the test object, and the result is similar to that of the implanted human body. However, it is difficult to distinguish the influence of complex factors on the experimental results. The *in vitro* experiment refers to the direct study (material itself) or indirect study (material extract) study of the effect of material effect on the growth, differentiation, or activity of tissue cells (Declercq et al., 2004). *In vivo* experiments need to consider the complexity of biological systems, and it is difficult to analyze a single factor objectively. Therefore, *in vitro* experiments are more sensitive to distinguishing biotoxicity and its effects on tissues and cells. Some studies revealed that some medical materials depict mild toxicity *in vitro* experiments but show good biocompatibility *in vivo* experiments (Suggs et al., 1999; Xie et al., 2016). This difference may be because cytotoxic substances released by material degradation are attenuated or eliminated by the complex biological regulatory system of the subject, thus masking their toxic effects. Therefore, only *in vivo* experiments cannot fully reflect the biocompatibility of tested materials. *In vivo* and *in vitro* experiments can counteract the influence of complex factors in the body to some extent and more fully reflect the impact of materials on cells.

To detect the toxicity of Ti-B12 material, MC3T3-E1 cells were cultured with Ti-B12 material extract, and cell morphology and proliferation were detected. At the same time, MC3T3-E1 cells and Ti-B12 were co-cultured, and cell apoptosis was detected by flow cytometry. Compared to the Ti6Al4V group and the blank control group, Ti-B12 material did not cause abnormal growth and apoptosis of MC3T3-E1 cells and had no adverse effect on cell proliferation rate. These results indicate that Ti-B12 material has low toxicity and meets the toxicity requirements of biological materials.

The implantation of biomaterials is like a foreign object invasion or trauma to the body, so it is necessary to evaluate the local and systemic reactions of materials in contact with the body. We injected the saline extract of Ti-B12 material into the abdominal cavity of mice and observed them for five consecutive days. No adverse reactions such as diarrhea, cyanosis, decreased exercise, and weight loss was found in mice. Additionally, the skin irritation experiment was carried out by directly contacting Ti-B12 with rabbit skin. The intradermal irritation experiment was conducted by injecting the Ti-B12 saline and olive oil extract into rabbit skin. The above studies demonstrated no erythema, lump, or swelling of the skin at the contact and injection sites, as in the Ti6Al4V group and the blank control group, with a score of 0, indicating that Ti-B12 implantation would not cause rejection.

Currently, some metal ions in commonly used medical alloy materials have a certain toxic effect on the human body. For example, Cr and Co have greater cytotoxicity and are metal metamorphosis sources. Ni can limit the growth of fibroblasts but also may cause allergic reactions or cancer; Al is neurotoxic, and V may be teratogenic or carcinogenic. However, Ti, Nb, Zr, Mo, Sn, and Nb are non-toxic or low-toxic elements. Yamamoto et al. reported that the toxicity of Nb to osteoblasts and fibroblasts was very low (Yamamoto et al., 1999). Zr might significantly improve osteoblast adhesion (Sista et al., 2013), which is currently marketed as Roxolid (Straumann, Basel, Switzerland). Sn has been found non-toxic and non-allergic (Niinomi, 2003). Thus, Tin (Sn) seems to be an alloying element that is safe to use with Ti. In several previous studies, Moelements h shown good biocompatibility. The components of Ti10Mo6Zr4Sn3Nb alloy are all elements with good biocompatibility. The research results also present that the material has good biocompatibility and meets the safety standards of biological materials.

The osseointegration performance of orthopedic implant material refers to the bony fusion of material with surrounding bone without connective tissue growth, which can improve the binding force of the material-bone interface and ensure the long-term stability of the prosthesis. Osteoblasts are one of the most important cells in bone tissue repair. An osteoblast adheres to the surface of a material which is essential for osseointegration. Acridine orange cell staining can count the number of cells, distinguish normal growth, apoptotic, and necrotic cells, and depict the growth state of cells. The results of acridine orange cell adhesion assay revealed that when MC3T3-E1 cells were co-cultured with Ti-B12 alloy, osteoblasts could adhere to the surface of alloy, and the number of adherent cells was higher than that of Ti6Al4V alloy (*p* < 0.05), thus it can be proved that Ti-B12 alloy has better adhesion ability to osteoblasts.

Further verification of the adhesion of the Ti-B12 alloy to osteoblasts was performed using scanning electron microscopy to evaluate the osteoblast morphology adhering to the material surface. Most osteoblasts adhered to the material in the form of elongated spindles, with more pseudopodia, and the cell growth status was good, which was more than that of the Ti6Al4V group. These results indicated that Ti-B12 was more conducive to osteoblast adhesion.

ALP is an essential indicator for detecting the degree of osteoblast differentiation of osteogenic cells, which has a high content in bone tissue and plays a key role in the calcification process (Vimalraj, 2020). Osteoblast function can be evaluated by measuring ALP secretion and is also a specific index for evaluating tissue calcification ability and osteoblast activity (Nakamura et al., 2020). The ALP content test of osteoblasts demonstrated that osteoblasts in the Ti-B12 group secret more ALP than the Ti6Al4V and blank control groups (*p* < 0.05); this indicated that the alloy could enhance the secretion of ALP in osteoblasts. ALP was expressed in the process of formation and maturity of extracellular bone matrix expression, OCN expression in the bone matrix mineralization process is the main index of osteogenetic differentiation, and Runx2 is one of the important transcription factors for osteogenesis cell differentiation throughout the various stages of the directional differentiation process, the main control cell division cycle, and other transcription factors also play a role. This study illustrated that gene expression levels for ALP, OCN, and Runx2 demonstrated no significant difference between Ti-B12 group, Ti6Al4V group, and blank control group at 7 and 14 days after induction of differentiation. However, the Ti-B12 group was slightly higher than the latter two, indicating that Ti-B12 alloy has more advantages in promoting osteogenic differentiation.

Mineralized nodules are the final expression of the osteogenic phenotype of osteoblasts *in vitro* (Park et al., 2021) and are also characteristic indicators of mineralized matrix formation. Therefore, the study of terminal differentiation and functional status of osteoblasts can be achieved indirectly by detecting mineralized nodules. Calcium salts in mineralized nodules were stained dark red by alizarin red staining, and 10% hexapylpyridine chloride dissolved the red-stained mineralized nodules. Calcium ion content was proportional to absorbance value. Alizarin red staining of mineralized nodules demonstrated that there were larger and more dark red mineralized nodules in Ti-B12 group, which were higher in number and size than those in Ti6Al4V and blank control group. After solubilization, the OD values of mineralized nodules depicted that Ti-B12 group was significantly higher than Ti6Al4V and blank control group (*p* < 0.05). Ti-B12 can enhance the mineralization of extracellular matrix of osteoblasts and promote bone formation.

To further evaluate the integration of new titanium alloy Ti-B12 with the surrounding bone, we implanted it in rabbits and studied it by X-ray and histological analysis. In this study, X-rays displayed that the implant was in a good position and the surrounding tissues demonstrated no signs of bone density loss, demonstrating good osseointegration between the implant and the bone tissue. Additionally, hard tissue sections can be used to slice bone tissues containing metal materials. The tested materials do not need to be predecalcified, and the position relationship between implanted metal materials and bone tissues remains unchanged, which can reflect the growth status of implanted bone tissues without damaging the original tissue structure of implant-bone interface. After toluidine blue staining of hard tissue sections, it is possible to observe the growth of surrounding bone tissue, and the ability of the material to directly combine with bone tissue can be directly evaluated. In this study, both materials were wrapped in bone tissue, and there was no fibrous tissue space between the materials and the new bone. However, the binding rate between Ti-B12 and surrounding bone tissue was better than that of Ti6Al4V, which proved that Ti-B12 alloy with low elastic modulus was similar to the elastic modulus of human bone and could provide good stress conduction, thus promoting the formation and reconstruction of surrounding bone tissue.

There are some defects and deficiencies in this study: 1) Only MC3T3-E1 cells were used in the experiment, and several more cells were needed to evaluate the biocompatibility of the material in the follow-up; 2) This study only evaluated the biocompatibility and bone integration properties of the new titanium alloy materials prepared by forging process, which lacked the research on the properties of the new titanium alloy materials under different processing methods, and needed to be further supplemented by subsequent research.

## 5 Conclusion

β-titanium alloy material has broad application potential in orthopedics and maxillofacial surgery due to its excellent mechanical properties. The premise of its clinical transformation is excellent biocompatibility and osseointegration performance. According to this study, the new β-titanium alloy Ti-B12 has low toxicity, does not cause rejection reactions, and has better osseointegration performance than the traditional titanium alloy Ti6Al4V. Therefore, Ti-B12 material is expected to be further promoted in clinical practice.

## Data Availability

The raw data supporting the conclusion of this article will be made available by the authors, without undue reservation.
